# Study protocol for a randomised controlled trial of CBT vs antipsychotics vs both in 14–18-year-olds: Managing Adolescent first episode Psychosis: a feasibility study (MAPS)

**DOI:** 10.1186/s13063-019-3506-1

**Published:** 2019-07-04

**Authors:** Melissa Pyle, Matthew R. Broome, Emmeline Joyce, Graeme MacLennan, John Norrie, Daniel Freeman, David Fowler, Peter M. Haddad, David Shiers, Chris Hollis, Jo Smith, Ashley Liew, Rory E. Byrne, Paul French, Sarah Peters, Jemma Hudson, Linda Davies, Richard Emsley, Alison Yung, Max Birchwood, Eleanor Longden, Anthony P. Morrison

**Affiliations:** 1 The Psychosis Research Unit, Department of Psychology, Greater Manchester Mental Health NHS Foundation Trust, Prestwich, M25 3BL UK; 2 Division of Psychology and Mental Health, University of Manchester, Zochonis Building, Manchester, M13 9PL UK; 3 Institute for Mental Health, School of Psychology, University of Birmingham, Edgbaston, Birmingham, B15 2TT UK; 4 Department of Psychiatry, Medical Sciences Division, University of Oxford, Warneford Hospital, Oxford, OX3 7JX UK; 5 Centre for Human Brain Health, School of Psychology, University of Birmingham, Edgbaston, Birmingham, B15 2TT UK; 6 Oxford Health NHS Foundation Trust, Warneford Hospital, Oxford, OX4 7JX UK; 7 Centre for Healthcare Randomised Trials, Health Services Research Unit, University of Aberdeen, 3rd Floor Health Sciences Building, Aberdeen, AB25 2ZD UK; 8 Edinburgh Clinical Trials Unit, Centre for Population Health Sciences, Usher Institute, Nine Edinburgh BioQuarter, 9 Little France Road, Edinburgh, EH16 4UX UK; 9 School of Psychology, Pevensey Building, University of Sussex, Falmer, BN1 9QH UK; 10 Department of Psychiatry, Hamad Medical Corporation, PO Box 3050, Doha, Qatar; 11 NIHR MindTech MedTech Co-operative, Division of Psychiatry and Applied Psychology, Institute of Mental Health, University of Nottingham, Innovation Park, Triumph Road, Nottingham, NG7 2TU UK; 12 School of Allied Health and Community, Bredon Building, University of Worcester, Worcester, WR2 6AJ UK; 13 Centre for Educational Development, Appraisal and Research, University of Warwick, Coventry, CV4 7AL UK; 14 Forward Thinking Birmingham, Birmingham Women’s and Children’s NHS Foundation Trust, Finch Road, Lozells, B19 1HS UK; 15 Institute of Psychology, Health and Society, University of Liverpool, Waterhouse Building, Liverpool, L69 3BX UK; 16 Division of Population Health, Health Services Research & Primary Care, University of Manchester, Jean MacFarlane Building, Manchester, M13 9PL UK; 17 Department of Biostatistics & Health Informatics, Institute of Psychiatry, Psychology & Neuroscience, King’s College London, Denmark Hill, London, SE5 8AZ UK; 18 Centre for Youth Mental Health, Faculty of Medicine, University of Melbourne, 35 Poplar Rd, Parkville, Melbourne, VIC 3052 Australia; 19 Warwick Medical School-Mental Health and Wellbeing, University of Warwick, Coventry, CV4 7AL UK

**Keywords:** First-episode psychosis, Cognitive behavioural therapy, Family intervention, Psychological intervention, Antipsychotic medication, Adolescent psychosis, Schizophrenia, Randomised controlled trial

## Abstract

**Background:**

Adolescent-onset psychosis is associated with more severe symptoms and poorer outcomes than adult-onset psychosis. The National Institute for Clinical Excellence (NICE) recommend that adolescents with first episode psychosis (FEP) should be offered a combination of antipsychotic medication (APs), cognitive behavioural therapy (CBT) and family intervention (FI). The evidence for APs in treating psychosis is limited in adolescents compared to adults. Nevertheless, it indicates that APs can reduce overall symptoms in adolescents but may cause more severe side effects, including cardiovascular and metabolic effects, than in adults. CBT and FI can improve outcomes in adults, but there are no studies of psychological interventions (PI) in patients under 18 years old. Given this limited evidence base, NICE made a specific research recommendation for determining the clinical and cost effectiveness of APs versus PI versus both treatments for adolescent FEP.

**Methods/design:**

The current study aimed to establish the feasibility and acceptability of conducting such a trial by recruiting 14–18-year-olds with a first episode of psychosis into a feasibility prospective randomised open blinded evaluation (PROBE) design, three-arm, randomised controlled trial of APs alone versus PI alone versus a combination of both treatments. We aimed to recruit 90 participants from Early Intervention and Child and Adolescent Mental Health Teams in seven UK sites. APs were prescribed by participants’ usual psychiatrists. PI comprised standardised cognitive behavioural therapy and family intervention sessions.

**Discussion:**

This is the first study to compare APs to PI in an adolescent population with FEP. Recruitment finished on 31 October 2018. The study faced difficulties with recruitment across most sites due to factors including clinician and service-user treatment preferences.

**Trial registration:**

Current controlled trial with ISRCTN, ISRCTN80567433. Registered on 27 February 2017.

**Electronic supplementary material:**

The online version of this article (10.1186/s13063-019-3506-1) contains supplementary material, which is available to authorized users.

## Background

Psychosis is a mental health difficulty that encompasses a range of experiences including unusual or distressing beliefs and/or hallucinations, cognitive difficulties and disorganised speech and/or behaviour. In schizophrenia, a specific psychotic disorder, positive symptoms such as hallucinations and delusions can continue episodically for years and people often experience persisting disability partly due to negative symptoms (e.g. anhedonia and motivation loss) and cognitive difficulties (e.g. memory and attention deficits) [1, 2]. Findings suggest that adolescent-onset psychosis may be associated with more severe initial symptoms than adult-onset [3] and a more severe course; including greater functional impairment [4], poorer social outcomes and educational achievement [5], more days in hospital and greater readmission rates [6]. Adolescent-onset psychosis and schizophrenia is associated with significant societal costs and young people with psychosis and schizophrenia accounted for 25% of adolescent psychiatric inpatient admissions in England and Wales between 1998 and 2004 [7]. Access to efficacious, evidence-based interventions for young people with psychosis is therefore vital. In this paper, we will use the term “psychosis” to represent people both with and without a diagnosis of a schizophrenia spectrum disorder. Embracing diagnostic uncertainty is essential when working with people with a first episode of psychosis (FEP), as premature diagnosis may lead to pessimism and consequently poorer outcomes [8]; additionally, clients may perceive the term “psychosis” as more acceptable than a diagnostic label such as “schizophrenia” [8].

The National Institute for Health and Care Excellence (NICE) guideline for psychosis and schizophrenia in children and young people (CG155) recommends that children and young people (CYP) should be offered oral antipsychotic medication (APs) in conjunction with psychological interventions (family intervention (FI) with individual cognitive behavioural therapy (CBT)) [9]. If a young person and their parents wish to try psychological intervention alone, CG155 suggests that the care team should advise that psychological interventions (PI) are more effective when delivered in conjunction with APs, but offer individual CBT and FI if the young person and family wish to pursue that option [9]. However, CG155 also highlights that the evidence base available for these treatments in CYP is limited and relies on evidence derived largely from studies in adults.

A systematic review of pharmacological and psychological treatments for CYP with psychosis and schizophrenia identified 19 studies of APs [10]. Meta-analysis showed small effects of APs compared to placebo for positive and negative symptoms, depression and psychosocial functioning and large effects for global symptoms. However, data quality across the studies was considered poor [10]. A more recent meta-analysis of randomised controlled trials (RCTs) confirmed the efficacy of APs in CYP with psychosis but highlighted the limitations of the evidence base, especially on safety outcomes [11]. Antipsychotics are associated with a wide range of adverse effects including cardiovascular, metabolic, hormonal and extrapyramidal adverse effects [12]. Compared to adults, CYP may be more prone to developing antipsychotic adverse effects [13]. In particular, antipsychotic-associated weight gain is greater in FEP than in multi-episode schizophrenia [14]. A study by Corell et al. [15] showed that 12 weeks of antipsychotic treatment in children and adolescents, who had less than 1 week’s prior antipsychotic exposure, was associated with significant rates of obesity and new-onset categorical glucose and lipid abnormalities. For example, weight gain > 7% in the participants ranged from 56% (when participants were treated with quetiapine) to 84% (with olanzapine) [15]. To date, there are no studies evaluating the long-term safety of APs in adolescents with psychosis. Therefore, evaluating long-term cost-benefit ratios is not currently possible. For some adolescents with psychosis, the risks of APs may outweigh their benefits. Moreover, discontinuation of APs against medical advice and non-adherence are common in schizophrenia, especially in the first episode [16]. Together, these factors support the importance of assessing potential non-pharmacological treatment options for psychosis.

There are currently no trials of CBT or FI with CYP under 18 years old with psychosis [10]. There are seven low-quality studies of CBT and/or FI for psychosis in young people (aged between 15 and 24 years old). Meta-analysis of the data from these studies indicates no evidence of treatment effects on symptoms, and low-quality evidence for a combination of CBT and FI on the number of days to relapse [10]. There is better-quality evidence for the effectiveness of psychological interventions for psychosis from studies conducted in adults. Meta-analyses in adult populations suggest that a combination of CBT and APs has small but statistically significant effects on symptoms and rehospitalisation rates [17, 18]. FI has been shown to reduce relapse rates [19], and there is a signal that CBT alone can reduce symptoms in adults who choose not to take APs, particularly in participants under 21 years old [20]. The COMPARE trial [21], which allocated people aged 16+ years with FEP to receive either APs, CBT or a combination of both, found no differences in Positive And Negative Syndrome Scale (PANSS) scores between the AP-only and the CBT-only group, or between the combination group and the AP-only group at 12-months. However, PANSS scores were significantly lower in the combination group than in the CBT-only group at 12 months. Those in the CBT-only group reported fewer non-neurological side effects than those in the AP-only and combined groups [21], suggesting a potential for reduced adverse effects. A fifth of participants on the COMPARE trial (15 out of 75) were aged 16–18 years.

In summary, the NICE guideline (CG155) group determined that the available evidence, including that from adults, was sufficiently strong to recommend a combination of APs, CBT and FI as treatments for CYP with psychosis. However, for CYP with psychosis the balance of risks and benefits of APs appears less favourable and research is needed to establish the potential for psychological treatments, alone and in combination with APs, in this population. Consequently, CG155 recommended research to determine the clinical and cost effectiveness of psychological treatment alone, compared with antipsychotic medication and compared with psychological treatment and antipsychotic medication combined [9]. Subsequently, the National Institute for Health Research (NIHR), Health Technology Assessment Programme (HTA) put out a commissioned call (HTA 51/13) to answer this research question.

The Managing Adolescent first episode Psychosis Study (MAPS) is funded by the UK NIHR HTA programme to establish whether it is feasible and acceptable to run a definitive trial examining the effectiveness of antipsychotic monotherapy versus psychological intervention (CBT plus FI) versus a combination of these treatment options in adolescents with FEP. This will answer important questions about the feasibility and design of a definitive clinical and cost effectiveness trial as recommended by NICE guideline CG155.

## Methods/design

Our specific objectives were to assess (1) the proportion of eligible people that clinicians are willing to refer to the trial, and the proportions of eligible referred people that are willing to participate and to comply with their allocation; (2) the rates of adherence to follow-up assessments; (3) the characteristics of trial participants (to clarify selection criteria); (4) the feasibility and acceptability of the interventions to participants, parents and clinicians, and the appropriateness of treatment protocols; (5) the randomisation and masking procedures and (6) the validity and relevance of the measures to determine their acceptability, effectiveness and safety in a definitive trial. Furthermore, we aimed to estimate plausible ranges of sample size parameters to inform a definitive trial; finalise treatment manuals and outcome measures, and clarify training/supervision needs for delivering assessments and interventions; and assess the possibility for economies of scale, and monitor the research assistants’ time use.

This study is a prospective randomised open blinded evaluation (PROBE) design, feasibility, RCT, which aimed to recruit 90 participants with FEP. As a PROBE study, persons (research assistants) that were unaware of the randomisation allocation made decisions about the scoring of outcome measures. We did not have a blinded end-point committee, however, our independent Data Monitoring and Ethics Committee (iDMC) provided oversight of unblinding.

The randomisation ratio was 1:1:1, stratified by centre and family contact (as participants without regular family contact only received individual CBT and not FI, if randomised to the PI-only and combined groups). Randomisation at the individual level was independent and concealed, using random permuted blocks. Research assistants (RAs) performed the randomisation procedure via a study-specific website developed by the Centre for Healthcare Randomised Trials (CHaRT), the UK Clinical Research Collaboration (UKCRC) registered Clinical Trials Unit (CTU #7) supporting the study. Aside from the trial therapists, the Trial Manager and Chief and Principal Investigators received emails informing them of randomised treatment allocation, so they could monitor adherence to allocation and provide supervision to therapists. The trial administrator was also informed via the same methods, and sent letters to participants to inform them of their allocation. Participants’ care teams, including their treating psychiatrist, were also informed of allocation.

Trial RAs were blind to the participants’ allocations until all outcome measures were completed for all participants. Maintaining the outcome assessor blind is crucial for effectiveness outcomes and also throughout the trial for measuring safety. The iDMC and Trial Steering committee (TSC) regularly monitored unblinding by each centre, and were able to implement corrective action if necessary. RAs and therapists received rigorous in-house training on the importance of maintaining the blind and methods to achieve this, and were required to read and sign our standard operating procedure (SOP) for retaining and managing blinding. Methods outlined in the SOP included separate offices for the therapists and RAs, protocols for answering telephones, including reminders for clinicians, participants and family members about the blind, protocols for message taking and secretarial support, separate diaries, pigeon holes and databases, using passwords and encryption of randomisation information. Any accidental unblinding was recorded. The Chief Investigator (CI) reviewed all unblinding to determine any patterns.

We recruited participants from Early Intervention in Psychosis (EIP) teams and Child and Adolescent Mental Health Services (CAMHS) in UK National Health Service (NHS) Trusts across seven sites; Greater Manchester, Lancashire, Oxfordshire and Buckinghamshire, Sussex, Northumberland, Tyne and Wear, Norfolk & Suffolk, and Birmingham. We included 14–18-year-olds presenting with FEP who were under the care of EIP and/or CAMHS services and who were seeking help for their experiences. Participants had to be within a year of presentation to services with psychosis and score 4+ on the PANSS delusions and/or hallucinations subscale(s) for a minimum of seven consecutive days, to determine their first-episode status and current symptomology. They also needed to either meet International Classification of Diseases Version 10 (ICD-10) criteria for schizophrenia, schizoaffective disorder or delusional disorder (according to the diagnosis recorded by the treating psychiatrist) or entry criteria for an EIP service. Finally, participants had to be competent to provide written, informed consent and those under 16 years old had to have a parent/guardian willing to provide additional consent to contact their child (for ethical reasons). If allocated to a PI arm, participants chose whether they wished to engage in FI. If participants did not wish to engage in FI, they were not prevented from participating in the trial and receiving CBT. No research procedures were carried out with family members, i.e. we did not collect any data on them or from them.

We excluded people who had diagnoses of a moderate/severe learning disability, ICD-10 organic psychosis, or primary alcohol/substance dependence (to ensure that our population was representative of young people with a primary problem of FEP), those who could not speak English, who scored 5+ on the conceptual disorganisation item of the PANSS (to ensure that people had capacity to engage in assessments and talking therapy), those who presented with immediate risk to themselves or others, or who had received APs or structured PI within the last 3 months prior to referral (to ensure treatment naivety). We did not exclude those with autistic spectrum disorder (ASD).

The protocol is reported in adherence with the Standard protocol items: recommendations for interventional trials (SPIRIT) guidelines; see Additional file 1.

### Interventions

#### Psychological intervention

PI was delivered by appropriately trained therapists (clinical psychologists or other mental health professionals with relevant training in CBT for psychosis (CBTp)). Participants were offered up to 30 sessions of individual CBT over a 6-month treatment window on an approximately weekly basis, and the option of up to six sessions of FI. Both CBT and FI were informed by an integrative cognitive model of psychosis [22]. This therapeutic model has been used successfully in other clinical trials in young people with psychosis, and participants within these studies provided positive qualitative interview feedback about its acceptability and usefulness [23]. Therapists took an assertive outreach approach developed from youth work principles and adopted principles from social and vocational interventions (e.g., supported education and/or employment interventions).

The overall aims of CBT were to reduce distress (particularly that arising from psychotic symptoms) and improve quality of life. CBT involved assessment and psychological formulation of people’s problems and goals, to allow an individualised therapeutic approach whilst retaining standardised components and clear boundaries. CBT was collaborative, with therapist and client agreeing on problems and goals to work on, using interventions dependent on clients’ individualised formulations from a range described in our published manuals [24–26]. There were a range of treatment targets including positive symptoms, comorbid problems (including anxiety, depression and substance use) and social issues such as improving relationships, developing valued social roles and maintaining functioning.

There were 4 phases to the CBT intervention: (1) engagement, assessment and formulation of problems and goals; (2) formulation-derived change strategies enabling people to work towards their individual goals; (3) development of an historical formulation, focusing on vulnerability factors leading to the development of FEP and including self-esteem work and (4) maintaining wellness and preventing relapse.

FI was grounded in a psycho-educational model of family work based on the behavioural family therapy (BFT) approach [27]. An initial session included an assessment of family understanding and appraisals of presenting difficulties, sharing the emerging psychological formulation, and agreeing a problem list and family intervention goals. Further sessions included an educational component to develop a common understanding, providing normalising and recovery-orientated information on presenting difficulties, problem-solving, communication skills training and relapse prevention planning. Families were given between-session tasks and encouraged to hold family meetings to support skills practice. Family members were also given information about local services and signposted to support for themselves, where appropriate. The approach was flexible and multi-faceted in response to needs and concerns addressing specific issues relating to adolescent-onset psychosis, including diagnostic uncertainty; dealing with emotional reactions/feelings evoked by the onset of psychosis; helping families “grieve” while encouraging a sense of agency and hope for recovery. FI was delivered in tandem with individual CBT by the same therapist. A maximum of six FI sessions were offered, spread out across the 6-month therapy window to match the pacing and content of individual CBT sessions and respond flexibly to concerns of family members as they arose. The final session was offered in collaboration with the participant’s care co-ordinator to ensure continuity following trial involvement and ensure goals/strategies are shared and supported.

To ensure fidelity to the treatment protocol, therapists received initial training and weekly supervision. With consent, therapy sessions were audio-recorded, and a randomly selected sample of tapes (stratified by stage of therapy) rated using the Cognitive Therapy Scale – Revised (CTS-R) [28]. The tape rating process continued throughout the lifetime of the trial to guide supervision, ensure fidelity to the treatment protocol and enable corrective action to be taken if necessary. Therapists completed session records after each appointment to provide detail about session content (e.g., agenda items, change strategies used and homework tasks).

#### Antipsychotic medication

Participants allocated to receive APs in the monotherapy or combined arms of the trial received their prescriptions from their usual psychiatrist in their care team. The psychiatrist was asked to make prescribing decisions consistent with the NICE guideline CG155, which recommends that clinicians should jointly decide the choice of antipsychotic medication with the young person and their parents/carers. The decision about medication should include discussion about the possible benefits and side effects of each drug, and provision of age-appropriate information [9]. AP prescribing should also be accompanied by physical health monitoring [9].

Psychiatrists were encouraged to initiate AP treatment as soon as possible following randomisation into the study, and to maintain AP treatment preferably for 26 weeks, but for 12 weeks minimum. The decision about the type and dose of AP was made by the psychiatrist as per their usual practice, i.e. we did not ask them to choose from a pre-specified list of antipsychotic medications. Psychiatrists were free to change dose and type of AP in response to efficacy and adverse events. A member of the research team who was not blind to allocation screened participants’ medical records to collect details about AP prescription. Additionally, self-report data were provided by participants either via our web-based platform or if preferred, in paper format that was returned to a research team member who was not blind to allocation. Psychiatrists on the study were available for consultation if participants’ CAMHS or EIP psychiatrists wished to discuss AP prescription.

We did not restrict the treatment options offered by the care team as it would have been unethical to do so. Additionally, participants were eligible to receive mental health medications other than APs, and psychological therapies other than and including CBTp or FI, throughout the course of the trial. A member of the research team who was not blind to allocation screened participants’ medical records for information on concomitant therapies received. In addition, self-report data on concomitant therapies were provided by participants using the method described above. Data from participants’ medical records and self-report data provided important feasibility information about participants’ adherence to their randomised treatment allocation.

### Outcomes

We do not have a single primary outcome, as this is not meaningful for a feasibility study. Our key outcomes to inform a definitive trial are rates of referral, recruitment, therapy attendance, adherence to medication, and completion of follow-up appointments and questionnaires. The acceptability of treatment will be determined by measuring discontinuation rates and through data collected within a nested qualitative study (see below). We have specific red/amber/green progression criteria that have been agreed with our iDMC, TSC and funder. Green would mean progression to a full trial is possible without the need for any substantial changes to the design or the way it was delivered; amber would mean we may need more resource to recruit, and ways of improving retention and compliance; and red would mean there is substantial doubt that the definitive study is feasible at an affordable cost. These progression criteria will be reviewed by the iDMC and TSC at the end of the trial and will inform a recommendation for a definitive trial. The progression criteria to a future definitive trial are described below:

1. Recruitment ≥ 80% of planned (green), recruitment within 79–60% of planned (amber), recruitment < 60% of planned (red).
2. Retention of participants within the study with baseline and outcome assessments at primary end point (6 months, end of treatment) ≥ 80% of primary outcome completed (green), 79–60% of primary outcome completed (amber), < 60% of primary outcome completed (red).
3. Satisfactory delivery of adherent therapy to ≥ 80% of groups receiving PI (green), 79–60% of groups receiving PI (amber), < 60% of groups receiving PI (red). Satisfactory delivery of adherent therapy is operationalised as attending 6 or more sessions of CBT.
4. Satisfactory delivery of antipsychotic medication to ≥ 80% of groups receiving AP (green), 79–60% of groups receiving AP (amber), < 60% of groups receiving AP (red). Satisfactory delivery of antipsychotic medication is operationalised as any exposure of AP for six consecutive weeks (this would include a dose below British National Formulary (BNF) lower limits given this is a frequent clinical practice for people of this age, and APs are licensed for adults).

**Fig. 1:**
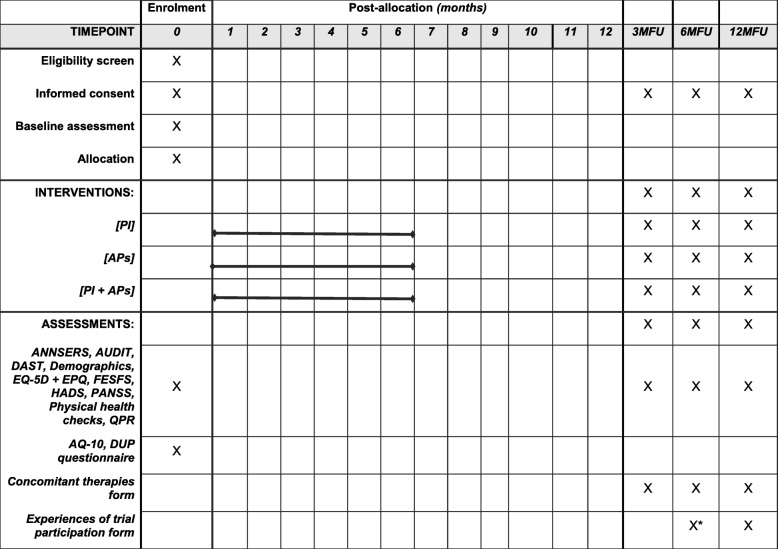
Schedule of enrolment, interventions and assessments. APs, antipsychotic medication; ANNSERS, Antipsychotic Non-Neurological Side Effects Scale; AQ-10, Autism Spectrum Quotient 10-item version; DAST, Drug Abuse Screener Test; DUP, duration of untreated psychosis; EPQ, Economic Patient Questionnaire; EQ-5D, EuroQol five dimension scale; FESFS, First Episode Social Functioning Scale; HADS, Hospital Anxiety and Depression Scale; PANSS, Positive And Negative Syndromes Scale; PI, psychological intervention; QPR, Questionnaire about the Process of Recovery; 3MFU, 6MFU, 12MFU 3-month, 6-month, 12-month follow up. *Only for participants who were randomised after the first 10 months of the trial and thus were not offered a 12MFU

We have a number of secondary outcomes, which were collected from participants via self-report and interview measures at baseline and follow-up appointments (see Fig. 1 for schedule of enrolment, interventions and assessments). This is to assess the acceptability and usefulness of the measures for inclusion in a definitive trial, rather than to measure the relative safety/efficacy of the interventions. Our provisional choice of primary outcome measure for a definitive trial is total PANSS score, which would ensure comparability to other antipsychotic and psychological therapy trials; however, a final decision on a primary outcome will need to be informed by data from this trial, associated qualitative studies and stakeholder opinion. The PANSS is a 30-item rating scale for psychopathologic assessment of adults with a diagnosis of schizophrenia [29]. It is a frequently used outcome measure in psychosis research. We assessed social and educational/occupational functioning with the First Episode Social Functioning Scale (FESFS) [30], a questionnaire developed with people presenting with FEP that has good reliability, validity and sensitivity to change. We measured subjective recovery using the 15-item version of the Questionnaire about the Process of Recovery (QPR) [31], a questionnaire developed in collaboration with people with psychosis. We used the Specific Psychotic Experiences Questionnaire (SPEQ) [32] to assess paranoia, hallucinations, cognitive disorganisation, grandiosity and anhedonia.

To measure common comorbidities, we used (1) the Hospital Anxiety and Depression Scale (HADS) [33], which has shown to be reliable and valid in measuring anxiety and depression symptoms over the past 7 days; (2) the Alcohol Use Disorder Identification Test (AUDIT) [34] and (3) the Drug Abuse Screening Test (DAST) [35]. The latter two measures are statistically predictive of the respective substance misuse disorders, using the Structured Clinical Interview for the Diagnostic and Statistical Manual version 5 (DSM-IV) (SCID-IV) [36]. We also measured diagnostic symptoms for autism spectrum conditions at baseline using the NICE-recommended 10-item version of the Autism Spectrum Quotient (AQ-10) [37]. We collected data on adverse effects of medication and trial participation (described further under “Safety Monitoring”). We collected basic health economics data about services used by participants, using an economic patient questionnaire adapted from previous studies conducted by the authors [38, 39] and the EuroQol five dimension, five level scale (EQ-5D-5 L) health status questionnaire [40]. This information will inform the design of the economic component of a definitive trial.

We designed a variable-length follow-up period, whereby participants recruited in the first 10 months were offered assessments at 3, 6 and 12 months and those recruited thereafter were only offered assessments up to the end of treatment (6 months, the proposed timing for the primary outcome). RAs collected all outcome measures at baseline and at the 3-month, 6-month and 12-month follow-up assessments. RAs underwent in-house training in administering all the measures to establish sufficient inter-rater reliability, including watching and scoring role-play and videos. These training sessions occurred prior to RAs delivering assessments independently and on several occasions throughout the course of the trial, to prevent “rater drift”. Measures were administered in a specific order agreed by the trial manager and CI to prioritise the most important (beginning with the PANSS).

To promote retention to the trial and reduce participant burden, RAs worked in a person-centred manner and gave participants control where possible over the location and time of appointments, offered breaks and multiple visits to complete measures, and gave the option of skipping measures if participants found them difficult to complete. Participants were compensated £10 per assessment and RAs contacted participants between follow-up appointments with a “check-in” phone call and a £5 shopping voucher. These processes are consistent with systematic review evidence for increasing retention in clinical trials [41]. RAs received weekly trial management supervision to enable monitoring of follow-up rate retention and to proactively problem-solve issues with completing assessments.

### Safety monitoring and reporting

We took a rigorous approach to recording and reporting serious adverse events. We recorded all serious adverse events (SAEs) at each point of contact with participants after randomisation. Our definition of an SAE was informed by the standard Health Research Authority definition and our trial protocol, which included all deaths, life-threatening incidents including suicide attempts, serious violent incidents, hospital admissions including admissions to secure units (we record whether these are voluntary or involuntary) and physical health units, prolongation of hospitalisation, any events resulting in persistent or significant disability or incapacity, any event consisting of a congenital abnormality or birth defect and formal complaints about treatment.

As noted, we scrutinised any instances of participants being admitted to psychiatric hospitals throughout the trial. The therapists or RAs were likely to have become aware of adverse events or admissions to psychiatric hospitals; however, we also screened each participant’s medical notes to assess for adverse events. All SAEs were monitored by the iDMC and TSC. All related and unexpected SAEs were reported to the National Research Ethics Committee (NREC) and the participant’s NHS Trust. We measured potential adverse effects associated with trial participation at the participant’s final follow-up assessment (i.e. 6 months or 12 months, dependent on when they were randomised into the trial), using a measure developed in our HTA-funded FOCUS trial [42]. In addition, if a participant in the monotherapy arm experienced a substantial deterioration in their mental health throughout the study, they were offered a switch to the combined treatment arm. This would be offered if the participant’s mental state had significantly deteriorated from baseline at the 3-month follow-up appointment (operationalised by an increase of 12.5% or more in rescaled PANSS scores) or if they were admitted involuntarily to hospital at any point in the trial.

We measured adverse effects of medication; firstly by interviewing participants using the Antipsychotic Non-Neurological Side Effects Scale (ANNSERS) [43], and secondly by measuring weight, height, waist circumference and blood pressure. In addition, a blood sample was taken for assessment of fasting plasma glucose (FPG), glycated haemoglobin (HbA1C), serum prolactin levels, and lipids (total cholesterol, low-density lipoproteins (LDL), high-density lipoproteins (HDL) and triglycerides). ANNSERS, physical examinations and blood tests were conducted at baseline and at the 3-month, 6-month and 12-month assessment. The results of blood tests were sent to participants’ psychiatrists (or other responsible clinician) at each time point. Finally, during screening of participants’ medical records for SAEs as described above, we recorded any adverse events related to antipsychotic medication (i.e., side effects) that were reported in participants’ notes.

### Consent

RAs were responsible for consenting participants into the trial under the supervision of the Chief and Principal Investigators. In addition to receiving Good Clinical Practice training, RAs completed the online module “Informed Consent in Paediatric Research” offered by the NIHR. Parents/legal guardians and potential participants had at least 24 h to read the Research Ethics Committee (REC)-approved participant information sheet, before meeting with an RA for the informed consent visit. If the young person was under 16 years old, RAs were required to obtain written informed consent from their parent/legal guardian to contact the young person prior to taking written informed consent to participate from the young person. The RAs followed the same procedure as above to ensure parents/guardians were fully informed and understood the study information, and if satisfied, the RA requested the parent to sign an assent form allowing the RA to contact their child about the study. The RA could then arrange to meet with the young person to sign their own consent form. Ongoing consent was confirmed at each research assessment and documented in the participant’s research notes.

### Trial oversight

The Sponsor of the trial is Greater Manchester Mental Health NHS Foundation Trust. The Sponsor was responsible for auditing the conduct of the trial. Independent oversight of the trial was provided by the iDMC and TSC. The iDMC was composed of an independent chairperson, statistician, clinician and service user. The role of the iDMC was to monitor recruitment of study participants, ethical issues of consent, quality of data (including missing data), the incidence of adverse events, unblinding and withdrawals and any other factors that might have compromised the progress and satisfactory completion of the trial. The iDMC met on a 6-monthly basis. A copy of the iDMC charter was retained by the Trial Manager in the site file.

The TSC was composed of an independent chairperson, statistician, clinician and service user along with non-independent members; the CI, trial manager, a representative of the funder (NIHR HTA) and a representative of the Sponsor. The TSC met every 6 months to monitor and supervise progress, and consider reports and recommendations. Meetings occurred approximately 2–4 weeks after the iDMC so the iDMC report could inform the TSC meeting. Prior to each iDMC and TSC meeting, the independent members were required to declare any conflicts of interest. The outcome of each iDMC and TSC meeting was reported to the funder via the minutes of the meeting. The trial management group, which comprised grant applicants and other local investigators met on an approximately monthly basis to provide management oversight of recruitment, attrition, adverse events, blind breaks, withdrawals and data management. Following the addition of three more sites in May and June 2018, individual site management meetings were organised on a monthly basis to ensure oversight of site-specific issues.

### Data management

All of the information collected about the participants is strictly confidential. All participants’ personal identifiable data (PID) are kept securely in databases, on secure NHS computer drives that are only accessible to the research team and are protected by a password known only by study staff. All RAs’ computers are also individually password-protected. Paper copies of research data (i.e. assessment packs) are anonymised with a trial identification (ID) number and kept in a locked, filing cabinet separate to the storage of PID (such as referral forms and letters about the participant). Participants were made aware that although their data are strictly confidential and not shared outside of the research team, confidentiality could be broken in cases where participants were deemed to pose a risk to themselves or to others.

All research data entered on the secure web-based platform created by CHaRT were completely anonymised. To ensure the accuracy of the data entry for the proposed primary outcome measure (PANSS), RAs checked entries for every participant by comparing PANSS scores in the paper assessment files against the scores entered into the CHaRT platform. Data were checked once all possible assessments for each time point were completed. An error rate of 2% or less was deemed acceptable. If the error rate had been above 2% the trial statistician and methodologist would have advised on further data checking, although this was not necessary due to the accuracy of the data entry.

The final trial dataset is managed and held by our CTU, CHaRT. Requests for access to the dataset will be considered in the first instance by the CI and then the CTU.

### Sample size

We proposed a sample size of 90 participants across all sites (30 per treatment arm). The target sample size is sufficient to attain reliable sample size estimates [44], gain feasibility information about trial proceures and facilitate a power calucalation. No power calculation was performed for this study as the focus of feasibility study analysis is not hypothesis testing [45]. We will estimate 95% confidence intervals to indicate likely intervention effects for a definitive trial.

### Recruitment

We had a team of highly motivated research staff who were experienced in recruiting participants into clinical trials, working across carefully selected NHS sites with which we have successfully collaborated on previous trials. Many of our sites had established relationships with EIP and CAMHS teams due to having research staff embedded within the teams, and/or due to recruitment efforts in previous trials. All sites covered extensive geographical areas. We used a joint-working approach between research and clinical teams in each site to build positive relationships with clinical staff, identify all potential cases and encourage clinical staff to discuss the trial with service users to enhance the referral rate. This included regular contact between the RAs and the clinical teams. Supervision and team meetings allowed RAs to provide feedback on teams they were struggling to contact so that the team could collectively problem-solve ways of moving forward.

To ensure fidelity to the allocated treatment, we endeavoured to work closely with EIP and CAMHS psychiatrists. The exclusion criterion of AP treatment naivety meant that engaging those who prescribe to CYP with psychosis was an important aspect to recruitment. The trial design required the participant’s usual psychiatrist (within their care team) to prescribe an AP as soon as possible after randomisation, if the participant was allocated to the AP monotherapy or combined treatment arms. To engage EIP and CAMHS psychiatrists we ran continuing professional development (CPD)/training events, and the CI directly contacted psychiatrists to ask for their support. Additionally, NIHR-funded clinical studies officers (CSOs) within many sites had existing relationships with teams and helped promote the study, identified research-motivated staff and where possible reviewed team caseloads for potential MAPS candidates.

MAPS began recruiting in April 2017 as a four-site trial with a 15-month recruitment window (projecting 1.5 randomisations per month per site). The actual versus target site-recruitment varied considerably between sites. Barriers to recruitment were identified across all sites. In some instances, there was a strong treatment preference expressed by clinicians, young people and/or parents, which influenced referral to the trial and/or young people agreeing to take part. The incidence rates of young people either being referred to, or accepted into EIP teams were relatively low across all sites, although numbers did vary by site. We also found variation in the inclusion/exclusion criteria of EIP services both across and within sites, particularly in terms of applying diagnostic uncertainty to people’s experiences. For example, some teams did not accept people whose psychotic experiences were judged a product of mood, trauma or personality-related difficulties, or where the duration of untreated psychosis reached a particular threshold. As a result, in some instances this led to a discrepancy between young people’s eligibility for MAPS and for an EIP service. As AP prescription was made by the participant’s usual psychiatrist, entry into the trial was dependent on the young person’s psychiatrist agreeing that randomisation to either or both MAPS treatments would be appropriate. The discrepancy between MAPS inclusion criteria and individual EIP inclusion criteria therefore further limited the already small number of young people who were potentially eligible for the trial. Additionally, due to the necessity of psychiatrists agreeing to young people entering the trial (including not prescribing APs for them prior to trial entry as described above), engaging psychiatrists with positive views of the trial embedded within EIP/CAMHS teams was crucial for recruitment. This was not possible across all services due to clinicians’ individual views and practices in relation to the treatment of FEP in young people. Another factor that proved challenging to recruitment was the relatively large proportion of young people who had already been prescribed APs prior to initial contact with EIP/CAMHS services (for example, via care pathways involving crisis/home treatment teams or inpatient services).

The most successfully recruiting MAPS site engaged psychiatrists embedded within EIP who regularly conducted service assessments, and could therefore provide an opinion on whether there was clinical equipoise for a young person early into their accessing services (before prescription of APs is made). In order to evaluate the feasibility of replicating this model approach to recruitment we added three new sites (Birmingham, Norfolk & Suffolk and Northumberland, Tyne and Wear) in May 2018, and extended our recruitment window by four months to 31 October 2018.

### Data analysis

The main aims of the feasibility trial will be delivered both via the continued monitoring of descriptive data and the analysis of data following the last follow-up assessment. Analysis is ongoing and began after full recruitment and follow up (i.e. there were no interim analyses for efficacy, although the iDMC monitored trial progress and any safety issues on a regular basis).

The main analyses are based on an intention-to-treat approach, using all randomised participants. Since safety and unwanted effects should be analysed on the basis of the most accurate information, these analyses are based on treatment received rather than as-randomised. Treatment received for the analysis of safety and unwanted effects is defined as any dose of an antipsychotic prescribed by the participant’s responsible psychiatrist and any dose of CBTp or FI from a trial therapist. We will use descriptive statistics to summarise the key indicators of success of the trial, including participant recruitment; checks for absence of selective recruitment of participants; baseline balance and participant flow. We will report data in line with the consolidated standards of reporting trials (CONSORT) 2010 extension statement for pilot and feasibility trials [46]. Important summary statistics will be the number of participants referred through case managers and mental health staff, number of referrals found to be eligible and number of consenting individuals and recruited individuals to each arm. Numbers for discontinuation from the allocated interventions, withdrawal of consent and failure to provide follow-up outcome data will also be generated. We will also report the proportion of participants who received their allocated intervention compared to the proportion who did not, and the proportion of participants offered a move to the combined therapy arm due to deterioration.

We will report our feasibility results (recruitment, retention, adherence) overall, in order to inform decisions about the viability of a future definitive trial. However, we will also report our descriptive results and 95% confidence intervals on outcome measures by group. We will describe actual treatment received and treatment compliance (to account for departures from the randomised interventions). We will also report descriptive statistics for the components of psychological intervention received, including number of sessions and milestones achieved.

To inform a phase III trial we will conduct the following analysis to ensure the data conform to the assumptions of the tests that will be conducted at that stage: measures proposed as the primary (PANSS) and secondary outcomes (QPR) for the phase III study will be analysed by analysis of repeated measures using a mixed effects model to account for the discrete timing of the follow-up assessments. The presentation of the analysis will focus on point estimates and associated 95% confidence intervals rather than statistical significance (*p* values). Further analysis will test the correlation of each measure across all time points and the variation within the proposed outcome measure (mean and standard deviation) to inform a definitive sample size calculation for a phase III trial. No formal analysis will be performed to account for missing data as MAPS is a feasibility study.

We will measure within trial and also explore the literature on the possible effects of clustering by therapist and site. We anticipate that the chance of clustering in relation to drug outcomes will be negligible, given that we are expecting prescribing to follow NICE (CG155) guidance, although we will measure this in case of significant differences in prescribing practices between sites. We will adjust for site in analyses (therapist will be nested within site) and examine intraclass correlation coefficients to inform a plan for managing any such impact on design and analysis of a definitive trial.

All statistical analyses are pre-specified in a comprehensive statistical analysis plan (SAP) authored by the study statistician and agreed with the iDMC and TSC.

### Intervention and trial acceptability: qualitative interviews

A nested qualitative study aimed to identify key themes associated with the acceptability of the trial and interventions amongst CYP, family members/carers and clinicians. Individual semi-structured interviews explored participants’ subjective experiences of recruitment, random allocation and receiving interventions, including views of adverse effects and benefits; sought to identify barriers and solutions to participation; and ultimately aimed to identify themes relating to all of these issues. This will inform the design of a definitive trial and help further refine intervention, recruitment and retention procedures. We sought a maximum variance sample on key variables among participants (gender, age, ethnicity, site, engagement with interventions). The CYP interviews were conducted with participants from all treatment arms within the trial after 6 month assessments; this allowed us to explore people’s experiences of receiving the treatments and of participating in the trial. Based on our previous work we expected thematic saturation would be achieved within 15–20 CYP interviews [47], 15–20 family members/carers [48] and 15–20 clinicians. All interviews were audio-recorded and transcribed verbatim at which point any identifying information (names and places) was removed. Data are currently being thematically analysed [49]. This method results in a rich and accessible account of qualitative data through the researcher making sense of the data and reporting themes that emerge [49]. Data were coded systematically and iteratively, and were organised within Nvivo qualitative data analysis Software version 11 [50].

### Dissemination

The results from this study will be published in a peer-reviewed journal for dissemination amongst researchers and clinicians. We will follow the International Committee of Medical Journal Editors (ICMJE) recommendations for authorship and review these for each individual publication. The results will also be disseminated to participants, if they agree to this. Participants leaving the trial were asked whether they would like the results of the study and their preferred method for receiving them (e.g. post, telephone, email). The results will also be disseminated amongst the healthcare professionals and teams that have helped support recruitment for the trial.

## Discussion

Our trial is the first of its kind, as we are not aware of any other feasibility trials that have recruited young people with FEP into a three-arm RCT of AP monotherapy, PI monotherapy or a combined treatment. Our trial has a number of strengths including using methods to minimise selection bias, such as generation of randomised permuted blocks via a computer system and centralisation of allocation via a web-based platform. We adhered to detailed operational procedures to minimise detection bias as outlined in our SOP for maintaining the blind. We have a low risk of selective reporting as we have published our SAP on the CTU website following agreement by our iDMC and TSC. We used a rigorous approach to recording and reporting SAEs, including reports from trial staff and medical record screening for details of SAEs, as specified in our iDMC-approved SOP for SAEs. Moreover, we go beyond UK Health Research Authority (HRA) requirements for review of SAEs and in addition to review by the CI, the iDMC chairperson provided an independent review of whether each SAE is trial-related or not. In addition, our trial included a bespoke measure of potentially unwanted effects of trial participation.

In summary, APs and psychological interventions (PI), specifically, family intervention and cognitive behaviour therapy are recommended treatments for FEP in adolescents but evidence about relative efficacy/acceptability is limited. If PI were non-inferior to APs, within an acceptable margin, this could be a major advance in treating a vulnerable group with high sensitivity to APs. The MAPS trial provides crucial data on the feasibility and acceptability of conducting a phase III trial of these treatments and the conditions that would be required to conduct such a trial.

## Trial status

Recruitment began on 1 April 2017 and ended on 31 October 2018 with a total of 61 participants randomised into the trial across six of the seven sites: 25 in Oxfordshire and Buckinghamshire, 21 in Greater Manchester, 9 in Lancashire, 4 in Sussex, 1 in Northumberland, Tyne and Wear and 1 in Birmingham. This represents 67.8% of the original target of 90 participants. All PI sessions were finalised across all sites by 30 April 2019. All follow-up assessments were completed and data entered by 2 May 2019. Qualitative interview recruitment commenced on 1 September 2017 and finished on 22 January 2019. Statistical and qualitative analyses are currently ongoing. This paper represents version 5 (17 July 2018*)* of the MAPS protocol. All Principal Investigators, the REC and HTA were informed of protocol modifications.

## Additional file

Additional file 1: SPIRIT 2013 checklist: recommended items to address in a clinical trial protocol and related documents. (DOC 121 kb)

## Data Availability

Not applicable.

## Abbreviations

ANNSERS: Antipsychotic Non-Neurological Side Effects Scale
APs: Antipsychotic medication
AQ-10: Autism Spectrum Quotient 10-item version
ASD: Autistic spectrum disorder
AUDIT: Alcohol Use Disorders Identification Test
BFT: Behavioural family therapy
BNF: British National Formulary
CAMHS: Child and Adolescent Mental Health Services
CBT: Cognitive behavioural therapy
CBTp: Cognitive behavioural therapy for psychosis
CG155: Guideline for psychosis and schizophrenia in children and young people
CHaRT: Centre for Healthcare Randomised Trials
CI: Chief Investigator
CONSORT: Consolidated standards of reporting trials
CPD: Continuing professional development
CSO: Clinical Studies Officer
CTS-R: The revised Cognitive Therapy Scale
CTU: Clinical Trials Unit
CYP: Children and young people
DAST: Drug Abuse Screener Test
DSM-IV: Diagnostic and Statistical Manual version 5
EIP: Early intervention in psychosis
EME: Efficacy and Mechanism Evaluation
EPQ: Economic Patient Questionnaire
EQ-5D: EuroQol five dimension scale
FEP: First episode of psychosis
FESFS: First Episode Social Functioning Scale
FI: Family intervention
FPG: Fasting samples of plasma glucose
HADS: Hospital Anxiety and Depression Scale
HbA1c: Glycated haemoglobin
HDL: High-density lipoproteins
HRA: Health Research Authority
HTA: Health Technology Assessment
ICD-10: International Classification of Diseases Version 10
ICMJE: International Committee of Medical Journal Editors
ID: Identification
iDMC: Independent Data Monitoring Committee
iTSC: Independent Trial Steering Committee
LDL: Low-density lipoproteins
MAPS: Managing Adolescent Psychosis Study
NCCMH: National Collaborating Centre for Mental Health
NHS: National Health Service
NICE: National Institute for Health and Care Excellence
NIHR: National Institute for Health Research
NREC: National Research Ethics Committee
PANSS: Positive And Negative Syndromes Scale
PI: Psychological intervention
PID: Patient identifiable data
PROBE: Prospective randomised open blinded evaluation
QPR: Questionnaire about the Process of Recovery
RA: Research Assistant
RCT: Randomised controlled Trial
REC: Research Ethics Committee
SAE: Serious adverse event
SAP: Statistical analysis plan
SCID-IV: Structured Clinical Interview for DSM-IV
SOP: Standard operating procedure
SPEQ: Specific Psychotic Experiences Questionnaire
SPIRIT: Standard protocol items: recommendations for interventional trials
TM: Trial Manager
TMM: Trial Management Group
TSC: Trial Steering committee
UKCRC: UK Clinical Research Collaboration
